# Uncovering codon usage patterns during murine embryogenesis and tissue-specific developmental diseases

**DOI:** 10.3389/fgene.2025.1554773

**Published:** 2025-05-26

**Authors:** Sarah E. Fumagalli, Sean Smith, Brian Lin, Rahul Paul, Collin Campbell, Luis Santana-Quintero, Anton Golikov, Juan Ibla, Haim Bar, Anton A. Komar, Ryan C. Hunt, Michael DiCuccio, Chava Kimchi-Sarfaty

**Affiliations:** ^1^ Hemostasis Branch, Division of Plasma Protein Therapeutics, Office of Tissues and Advanced Therapies, Center for Biologics Evaluation and Research (CBER), Food and Drug Administration (FDA), Silver Spring, MD, United States; ^2^ High-performance Integrated Virtual Environment (HIVE), Office of Biostatistics and Pharmacovigilance (OBPV), Center for Biologics Evaluation and Research (CBER), US Food and Drug Administration (FDA), Silver Spring, MD, United States; ^3^ Department of Anesthesiology, Critical Care and Pain Medicine, Boston Children’s Hospital and Harvard Medical School, Boston, MA, United States; ^4^ Department of Statistics, University of Connecticut, Storrs, CT, United States; ^5^ Center for Gene Regulation in Health and Disease, Department of Biological, Geological and Environmental Sciences, Cleveland State University, Cleveland, OH, United States; ^6^ Department of Biochemistry and Center for RNA Science and Therapeutics, School of Medicine, Case Western Reserve University, Cleveland, OH, United States; ^7^ Independent Researcher, Rockville, MD, United States

**Keywords:** mouse embryology, tissue-specific, transcriptomic-weighted, relative synonymous codon usage, clustering methods, disease-associated comparison, machine learning

## Abstract

**Introduction:**

Mouse models share significant genetic similarities with humans and have expanded our understanding of how embryonic tissue-specific genes influence disease states. By improved analyses of temporal, transcriptional data from these models, we can capture unique tissue codon usage patterns and determine how deviations from these patterns can influence developmental disorders.

**Methods:**

We analyzed transcriptomic-weighted data from four mouse strains across three different germ layer tissues (liver, heart, and eye) and through embryonic stages. Applying a multifaceted approach, we calculated relative synonymous codon usage, reduced the dimensionality, and employed machine learning clustering techniques.

**Results and discussion:**

These techniques identified relative synonymous codon usage differences/similarities among strains and deviations in codon usage patterns between healthy and disease-linked genes. Original transcriptomic mouse data and RefSeq gene sequences can be found at the associated Mouse Embryo CoCoPUTs (codon and codon pair usage tables) website. Future studies can leverage this resource to uncover further insights into the dynamics of embryonic development and the corresponding codon usage biases that are paramount to understanding disease processes of embryologic origin.

## 1 Introduction

Embryogenesis is a dynamic process that is orchestrated through precise spatiotemporal control of gene expression, affecting the growth, division, reorganization, localization, and differentiation of specific cell types (Tam and Loebel, 2007; Asp et al., 2019; Thompson et al., 2014; Kang et al., 2011). Disruptions to these sophisticated expression programs can lead to developmental diseases and tissue malformation (Ben-Porath et al., 2008; Zimmer et al., 2011; Barker, 2004). Recent insights into human embryology have largely been derived from studying developmental parallels within animal models, particularly embryonic mice (*Mus musculus*) (Shahbazi and Zernicka-Goetz, 2018). Additional insight has emerged from *ex utero* mouse embryogenesis (Aguilera-Castrejon et al., 2021) and complex genetic studies involving single-cell spatial transcriptomics of embryos (Tyser et al., 2021; Srivatsan et al., 2021; Pour and Yanai, 2022). These new systems have improved our understanding of tissue-specific gene function across developmental stages by providing access to large quantities of raw transcriptomic data on spatiotemporal variations in gene expression throughout embryonic stages (Theiler, 1972; Wong et al., 2015; Qiu et al., 2023; Cardoso-Moreira et al., 2019; Wang et al., 2021), which can be leveraged to gain further understanding of molecular processes underlying organogenesis and the etiology of developmental disorders.

Although the biological steps of developmental embryonic stages are mostly elucidated, it remains unclear what factors control the genetic programming of development and to what extent variations in the cell-state-specific transcriptome can impact healthy tissue development (Ren et al., 2007). In adult tissues, a tissue’s codon usage landscape can be substantially influenced by differential gene expression (Aguet et al., 2017; Shen-Orr et al., 2010). Synonymous codon usage is biased and derives from the degeneracy of the genetic code, whereby a set of 61 codons encodes for the 20 standard/common amino acids (AAs) used in protein synthesis. Codon usage bias (CUB) is present in genes, tissues, and organisms (Kames et al., 2020; Alexaki et al., 2019; Meyer et al., 2021), and some codons are determined to be “optimal” or “suboptimal” due to their capacity to influence mRNA stability and the variability in host tRNA concentrations, which can influence translational rates (Brule and Grayhack, 2017; Bae and Coller, 2022; Bali and Bebok, 2015). In most cases, genes requiring consistently high levels of expression contain more optimal codons to ensure stable and accurate translation of mRNAs (Bae and Coller, 2022; Hanson and Coller, 2018). Uddin (2024) found an overrepresentation of the valine codon GTG in all protein-coding genes related to Parkinson’s disease (Uddin, 2024). Divergent CUB in various tissues has been associated with numerous diseases (Meyer et al., 2021; Fornasiero and Rizzoli, 2019; Gun et al., 2017). For example, the conversion of an isoleucine (Ile) codon (ATC to ATT) associated with cystic fibrosis can introduce translational pauses causing changes in the mRNA structure and protein expression levels (Bartoszewski et al., 2010). A CUB skew toward AGG (arginine), as opposed to CTA (leucine, Leu), GTA (valine, Val), CAA (glutamine, Gln), and CGT (arginine, Arg), can be used as a gene editing target for the therapeutic treatment of neurodegeneration and cancer (Khandia et al., 2023).

Different organisms have exhibited dynamic transcriptome–proteome landscapes with distinct time-oriented gene expression profiles that affect the specification of embryonic tissue development (Shen-Orr et al., 2010; Allen et al., 2022). Alterations to mRNA transcriptomic patterns and associated codon usage have been proposed as a potential biomarker for many different diseases (Brule and Grayhack, 2017; Hanson and Coller, 2018; Malakar et al., 2016; Gillen et al., 2021), and therefore, characterizing CUB at various stages of embryonic development may yield an improved understanding of the molecular basis of congenital disorders. Rossi et al. (2022) found that biased codons are significantly more prevalent in disease-causing human genes, and this pattern is conserved across mammals (Rossi et al., 2022). However, most patients with congenital heart, liver, or eye disease do not carry any identifiable DNA mutations or chromosomal abnormalities attributable to structural disease (Moore-Morris et al., 2018; Portmann and Roberts, 2012; Francis, 2006). Variation in codon bias tends to show that a pattern of A/T-ending codons is expressed more coordinately across tissues and developmental stages than G/C-ending codons (Benisty et al., 2023). Increased understanding of normal/abnormal development will undoubtedly help better understand many risk factors for human birth defects and potentially allow for the development of novel strategies for both the prevention and treatment of these defects.

In this study, using publicly available sequencing data (Fumagalli et al., 2024), we identified distinct codon signatures between healthy and disease-associated genes throughout embryonic development. In addition, we also performed a comprehensive analysis of CUB in the developing liver, heart, and eye tissues through a variety of different clustering approaches and identified a unique set of synonymous codons characteristic for different strains and embryonic periods. This analysis pipeline represents the most comprehensive source of information on codon usage patterns across murine embryogenesis and can be effectively applied to future studies of developmental diseases.

## 2 Systems and methods

### 2.1 Mouse embryonic tissue samples: curation and quality control

**FIGURE 1:**
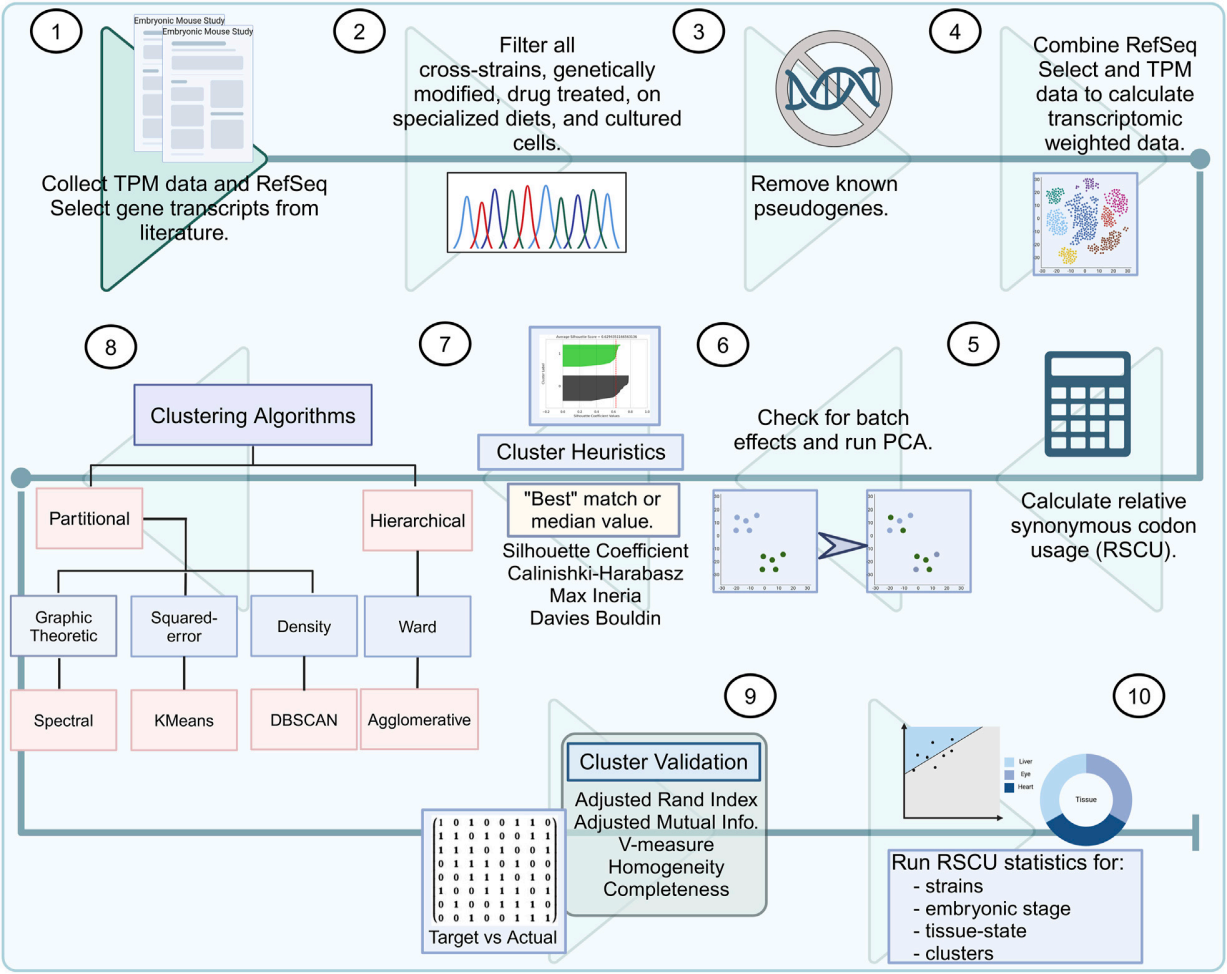
Murine embryonic data bioinformatics pipeline. (1) Literature research for bulk RNA-seq mouse embryo samples. (2) Quality control by removing cross-strain, genetically modified, drug-treated, or samples from mice on specialized diets. (3) All known pseudogenes should be removed from the transcripts per million (TPM) sample list and RefSeq select genes. (4) TPM counts should be used to weigh the codon usage of the RefSeq select genes. (5) The relative synonymous codon usage (RSCU) for all strains (C57BL/6, C57BL/6J, C57BL/6N, and CD-1) must be calculated across all embryonic stages (ranging from 6 to 18). (6) Batch effects must be checked among the strains, and the RSCU values should be used for all samples to calculate the principal component analysis (PCA). (7) Clustering heuristics must be used for determining the best-estimated number of clusters. (8) Embryonic data must be input through several clustering algorithms: Spectral, KMeans, DBSCAN, and agglomerative. (9) Evaluation metrics must be run for differences in the target groups and resulting clustering. (10) Finally, RSCU statistics must be calculated for strains, embryonic stage, tissue states, and clusters.

Details on data curation and quality control of the mouse embryo FASTQ files are discussed by Fumagalli et al. (2024) (Figure 1, steps 1, 2, and 3). Associated with these data is a user-friendly database, Mouse Embryo CoCoPUTs, which provides access to median GC content, codon, codon pair, dinucleotide, and junction dinucleotide usage values for the four strains discussed in this study (C57BL/6, C57BL/6J, C57BL/6N, and CD-1), 15 tissue types, 26 embryonic days (E), and 18 Theiler stages (https://dnahive.fda.gov/hivecuts/mouse_embryo/).

### 2.2 Tissue-specific gene filtering and transcriptomic gene weights

**FIGURE 2:**
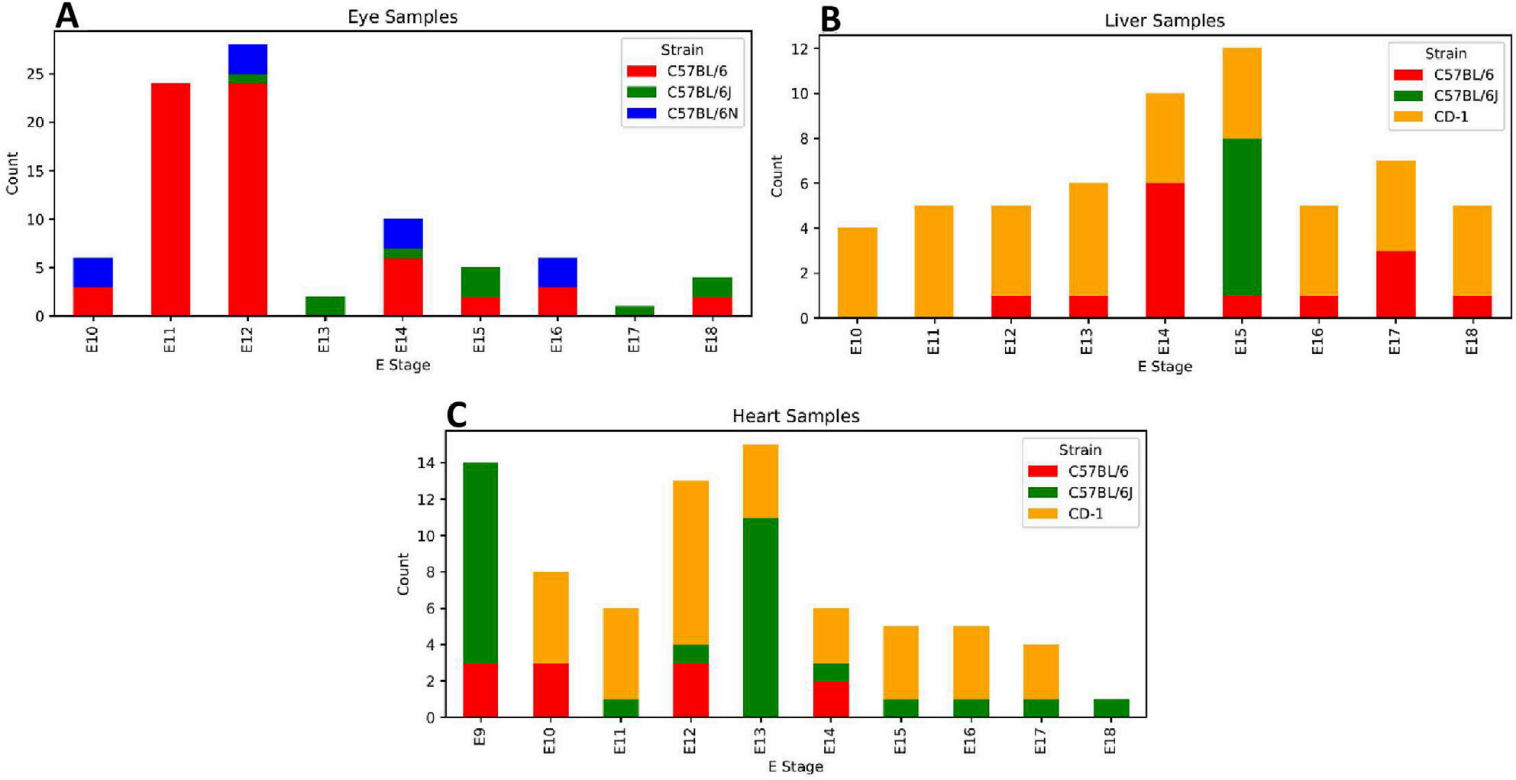
Embryonic stage (E) sample count per strain and tissue type. Each sub-plot contains a corresponding number of stacked bar graphs as E stages that describe the count of each strain for a specified tissue type. Strain colors: red, C57BL/6; green, C57BL/6J; blue, C57BL/6N; and orange, CD-1. Tissue plots: **(A)** eye, **(B)** liver, and **(C)** heart.

Transcript per million (TPM) samples were filtered based on tissue type (ectoderm: eye, mesoderm: heart, and endoderm: liver) (Figure 2). To filter the genes, we identified genes associated with healthy and diseased tissues using expression data from the Jackson Laboratory Mouse Gene Informatics gene expression database (https://www.informatics.jax.org/marker). For the disease-associated genes, we selected protein-coding genes as the feature type and searched individually by eye, liver, and heart under the “Mouse phenotypes and mouse models of human disease” section. For the healthy- or normal-associated genes, we ran a standard search on their gene expression data. After collecting the tissue-specific genes, we removed known pseudogenes [as listed in the study by Fumagalli et al. (2024)] and duplicates and retained only those on the Mouse Embryo CoCoPUTs gene list (Supplementary Table S1).

Then, we normalized the transcriptome-weighted codon usage by 1,000 (Figure 1, step 4). In this analysis, we did not process diseased and healthy mice but rather compared healthy tissue samples, weighted by sets of genes associated with disease or healthy tissue. Any genes overlapping with our TPM and RefSeq genes were kept as tissue-specific weights (Supplementary Table S1). We compared weighted data for the following categories: 1) healthy liver vs. diseased liver, 2) healthy eye vs. diseased eye, and 3) healthy heart vs. diseased heart.

### 2.3 Relative synonymous codon usage and normalization

#### 2.3.1 Relative synonymous codon usage

Relative synonymous codon usage (RSCU) is a relative measurement of synonymous codon usage for a given amino acid. This calculation has previously been described in the literature using similar thresholds (1.5: “highly overrepresented” and 0.5: “highly underrepresented”) (Figure 1 step 5) (Sharp and Li, 1987; Sharp et al., 1986; Chen et al., 2021; Yu et al., 2021). The average and standard deviation are reported in Supplementary Table S2.

#### 2.3.2 Batch effects

Batch effects are systematic variations in data that arise from technical sources rather than biological differences. They can confound analysis, particularly in high-throughput sequencing data, and correcting for these effects is crucial for accurate downstream analysis. Batch effects were accounted for using the Python pyComBat library (Behdenna et al., 2021) (Figure 1 step 6). Batch effects were corrected by library, using identifiers from the NCBI Sequence Read Archive (SRR), European Genome-phenome Archive (ERR), and DDBJ Sequence Read Archive (DRR). The differences in the data extracted from these libraries can be found in Additional File 2 (Fumagalli et al., 2024). The most notable differences in data used from these three sources are the varying sequencing methods and data types.

#### 2.3.3 Dimensionality reduction

After removing the three stop codons (TGA, TAG, and TAA) and two codons that each encode a single amino acid—ATG (coding for Met) and TGG (coding for Trp)—we used principal component analysis (PCA) to reduce the dimensionality from 59 to 5 or fewer components (Figure 1, step 6). Data were transformed using the scikit-learn StandardScaler Python package, and the number of principal components (PCs) was determined using the cumulative explained variance (scikit-learn Decomposition Python package); the threshold was met when 80% of the variance of the original features (codons) was captured (Pedregosa et al., 2011). To focus our discussion, we highlight PCs with loading values greater than 0.2 or less than −0.2 that contribute more than one synonymous codon for a given amino acid. The PC values were evaluated, and the discussion was limited to values greater than five. Our justification for these cutoffs is based on our data results. The majority of the loading values were between ± 0.2, and the majority of the PC values were less than 5. We focused our discussion on values that fall outside these ranges.

### 2.4 Clustering heuristics and methods

#### 2.4.1 Clustering heuristics

Several heuristics were calculated using scikit-learn to identify an appropriate expected number of clusters (Figure 1, step 7) (Pedregosa et al., 2011). For each data analysis, four heuristics [namely, silhouette coefficient, Calinski–Harabasz score (maximum score determines the best-expected number of clusters), maximum inertia (with the optimal number determined by the “knee” point using the Python repository Kneed, version 0.8.5), and Davies–Bouldin score (better scoring clusters are further apart and less dispersed)] were calculated and compared. These metrics may report different “best” numbers of clusters. We did not prioritize one metric over another. Instead, if two or more heuristics agreed, then we selected that “best” number. If there were no two heuristics that agreed, we used the median value across the tools.

#### 2.4.2 KMeans

This is an unsupervised machine learning technique that uses a specified number of clusters to initiate partitioning (scikit-learn KMeans) (Figure 1, step 8) (Pedregosa et al., 2011). For each data analysis, five attempts and three iterations were run with 30% of a random sample to determine the “best centers.” These centers, along with the expected number of clusters, were used to set up a final run using all tissue samples.

#### 2.4.3 Spectral

This clustering method can be beneficial when the clusters are highly non-convex. The data are normalized (scikit-learn preprocessing normalize) before calculating the PCA (Figure 1, step 8) (Pedregosa et al., 2011). The number of neighbors is defined by dividing the total number of samples per data analysis by four. Two types of affinity were evaluated [“rbf” (Gaussian) and “near-neighbors” (Euclidean)], and the highest silhouette score was used for further analysis.

#### 2.4.4 DBSCAN

This is a density-based clustering method (scikit-learn DBSCAN) that requires the input of two parameters: epsilon (eps) and a minimum number of samples (Figure 1, step 8) (Pedregosa et al., 2011). Eps or epsilon, a maximum distance neighborhood measurement, can be estimated using the scikit-learn NearestNeighbors and KneeLocator functions. The minimum number of samples was determined as twice the number of PCA dimensions.

#### 2.4.5 Hierarchical

This is an agglomerative clustering method that uses linkage distance (SciPy cluster hierarchy linkage) (Virtanen et al., 2020) (Figure 1, step 8). The “method” parameter for this clustering technique was set to “ward” to reduce variance within each cluster, and a dendrogram was used to display relationships between embryo tissue samples.

### 2.5 Clustering validation

Clustering results with one or more clusters were evaluated based on performance using several similarity metrics—adjusted rand index (ARI), adjusted mutual information (AMI), and V-measure (normalized mutual information with the arithmetic averaging method) [range between 0 and 1 (uniform)] (Rosenberg and V-Measure, 2007). We also examined the homogeneity and completeness of the clusters (range between 0 and 1). These metrics highlight tissue-type clusters and were implemented using Python’s scikit-learn metrics (Figure 1, step 9) (Pedregosa et al., 2011). The clustering tool with the best separation between the embryonic day and the tissue state was chosen for further discussion. The outcome of each test for each tissue type can be found in Supplementary Table S7.

### 2.6 Statistics

We statistically compared RSCU between healthy and diseased tissues per mouse strain (Supplementary Table S2) and for each embryonic stage (Supplementary Table S3). We used Python’s (version 3.8) SciPy library (Virtanen et al., 2020) and Pandas (McKinney, 2010) to run an exact two-sided Mann–Whitney U test to find raw *p*-values for each of the tests performed (Figure 1, step 10). Adjusted *p*-values for multiple comparisons were calculated using the Bonferroni correction via the Multipletests function from statsmodels (v0.15.0). The stringency of the correction depends on the number of comparisons (N). The null hypothesis was rejected for adjusted *p*-values ≤0.05. If the *p*-value is less than the threshold, the null hypothesis is rejected. Significance was calculated between codons for resulting tissue clusters. The magnitude of the *p*-value effect size was determined by calculating Cohen’s *d*, with the assumption of unequal variances. Effect sizes can be “very small” (0–0.1), “small” (0.2–0.35), “medium” (0.36–0.65), “large” (0.66–0.9), and “very large” (>1).

### 2.7 Human comparison

Human genomic codon usage data were downloaded from TissueCoCoPUTs (Kames et al., 2020). These data were then converted into RSCU values, where a codon is overrepresented with a value of ≥ 1.5 and underrepresented with a value of ≤ 0.5 (Sharp et al., 1986).

### 2.8 Figure preparation

All figures were created using Matplotlib 3.5.1. (Hunter, 2007), seaborn, and BioRender.

## 3 Results

### 3.1 RSCU patterns in embryonic tissues across developmental stages

We performed a comparison across embryonic stages, spanning approximately from E6 to E18, for four different murine strains (namely, C57BL/6, C57BL/6J, C57BL/6N, and CD-1). We examined RSCU, a common method for qualifying CUB, for individual liver, heart, and eye samples. The chosen individual tissues are each representative of a developmental germ layer [endoderm (liver), mesoderm (heart), and ectoderm (eye)]. We observed differences in the identities of over- and underrepresented synonymous codons across mouse strains weighed by different sets of tissue-specific genes (Table 1). RSCU heatmaps and their observed significant differences can be found in Supplementary Figure S1 and Supplementary Tables S2,S3.

**TABLE 1:** Over- (>1.5) and underrepresented (<0.5) synonymous codons per tissue per murine strain.

Tissue	Strain	Overrepresented codon (>1.5)	Underrepresented codon (<0.5)
Liver	CD-1	Ala-GCC^a^, Arg-AGG, Gln-CAG, Ile-ATC^a^, Leu-CTG^a^, Ser-AGC, Ser-TCT, Thr-ACC^a^, Val-GTG^a^	Ala-GCG^a^, Gln-CAA, Ile-ATA^a^, Leu-CTA^a^, Leu-TTA^a^, Pro-CCG^a^, Ser-TCG^a^, Thr-ACG^a^, Val-GTA^a^
	C57BL/6J	Ala-GCC, **Arg-AGA** ^a^, Leu-CTG^a^, Ser-AGC, Thr-ACC, Val-GTG^a^	Ala-GCG^a^, ***Arg-CGT***, Ile-ATA^a^, Leu-CTA^a^, Leu-TTA^a^, Pro-CCG^a^, Ser-TCG^a^, Thr-ACG^a^, Val-GTA^a^
	C57BL/6	Ala-GCC^a^, Ala-GCT, Arg-AGG, Gln-CAG^a^, Gly-GCC, Ile-ATC^a^, Leu-CTG^a^, Pro-CCC, Ser-AGC, Ser-TCT, Thr-ACC^a^, Val-GTG^a^	Ala-GCG^a^, Gln-CAA^a^, Ile-ATA^a^, Leu-CTA^a^, **Leu-CTT**, Leu-TTA^a^, Pro-CCG^a^, **Ser-TCA**, Ser-TCG^a^, Thr-ACG^a^, **Val-GTT**, Val-GTA^a^
Heart	CD-1	Ala-GCC^a^, Gln-CAG^a^, Ile-ATC^a^, Leu-CTG^a^, Ser-AGC, Thr-ACC^a^, Val-GTG^a^	Ala-GCG^a^, Gln-CAA^a^, Ile-ATA^a^, Leu-CTA^a^, Leu-TTA^a^, Pro-CCG^a^, Ser-TCG^a^, Thr-ACG^a^, Val-GTA^a^
	C57BL/6J	Ala-GCC^a^, Gln-CAG^a^, Ile-ATC^a^, Leu-CTG^a^, Ser-AGC, Thr-ACC^a^, Val-GTG^a^	Ala-GCG^a^, Gln-CAA^a^, Ile-ATA^a^, Leu-CTA^a^, Leu-TTA^a^, Pro-CCG^a^, Ser-TCG^a^, Thr-ACG^a^, Val-GTA^a^
	C57BL/6	Ala-GCC^a^, Gln-CAG^a^, Ile-ATC^a^, Leu-CTG^a^, Val-GTG^a^	Ala-GCG^a^, Gln-CAA^a^, Ile-ATA^a^, Leu-CTA^a^, Leu-TTA^a^, Pro-CCG^a^, Ser-TCG^a^, Thr-ACG^a^, Val-GTA^a^
Eye	C57BL/6N	Ala-GCC^a^, **Arg-CGC** ^a^, Gln-CAG^a^, Gly-GGC^a^, Ile-ATC^a^, ***Leu-CTC***, Leu-CTG^a^, ***Phe-TTC*** *,* **Pro-CCC**, **Ser-TCC** ^a^, Ser-AGC, Thr-ACC^a^, Val-GTG^a^	Ala-GCG^a^, Gln-CAA^a^, Ile-ATA^a^, Leu-CTA^a^, Leu-TTA^a^, Pro-CCG^a^, **Ser-TCA**, Ser-TCG^a^, Thr-ACG^a^, Val-GTA^a^, ***Val-GTT***
	C57BL/6J	Ala-GCC^a^, Gln-CAG^a^, Gly-GGC, Ile-ATC^a^, Leu-CTG^a^, Ser-AGC^a^, Thr-ACC^a^, Val-GTG^a^	Ala-GCG^a^, Gln-CAA^a^, Ile-ATA^a^, Leu-CTA^a^, Leu-TTA^a^, Pro-CCG^a^, Ser-TCG^a^, Thr-ACG^a^, Val-GTA^a^
	C57BL/6	Ala-GCC^a^, Gln-CAG^a^, Ile-ATC^a^, Leu-CTG^a^, Ser-AGC, Thr-ACC^a^, Val-GTG^a^	Ala-GCG^a^, Gln-CAA^a^, Ile-ATA^a^, Leu-CTA^a^, Leu-TTA^a^, Pro-CCG^a^, Ser-TCG^a^, Thr-ACG^a^, Val-GTA^a^

^a^ Codon shared between healthy and disease-weighted samples. Italicized codons are found only in disease-weighted samples. Bold codons are strain-specific per tissue.

#### 3.1.1 Disease-associated liver (endoderm) and eye (mesoderm) genes exhibit unique codon usage preferences

For the liver, we compared C57BL/6, C57BL/6J, and CD-1 across E10 through E18 for changes in RSCU variation over time (Supplementary Figures S1A–C, respectively). We identified 13, 6, and 9 highly overrepresented codons and 12, 8, and 9 highly underrepresented codons for the C57BL/6, C57BL/6J, and CD-1 healthy liver samples, respectively. This is in contrast with the lower representation observed in diseased liver codons—6, 3, and 5 overrepresented codons and 9, 9, and 8 underrepresented codons in the respective strains. Uniquely represented codons across tissues can be found in C57BL/6 healthy liver samples [CTT (Leu), TCA (Ser), and GTT (Val)] and C57BL/6J healthy [AGA (Arg)] and diseased liver samples [AGA (Arg) and CGT (Arg)].

Along with the difference in the representation of codons, disease-associated liver genes demonstrate unique differences in CUB properties. Compared to healthy liver, disease-associated liver samples exhibit less variation, on average, in RSCU (Supplementary Figures S1D–F). In addition, RSCU between healthy and diseased liver genes are significantly different among synonymous codons. We found that 95% of synonymous codons showed significantly different RSCU values between healthy and diseased C57BL/6 samples (Mann–Whitney U test; *p*-value<0.05), and 90% of this variation came from E14 samples (Supplementary Table S3). Effect sizes were calculated, and most notable time points were E14 [medium effect: CTG (Leu; 0.38)] and E17 [large effect: AGC (Ser; 0.70) and CTG (Leu; 0.66)]. C57BL/6J E15 liver samples show that 78% of CUB is significantly different, but the effect sizes are very small for most codons [the highest showing a small effect for CTG (Leu; 0.21)]. The CD-1 liver samples possess 88% of codons showing a significant difference, with the E12–E18 embryonic stage range contributing the most to this variation. Codon AGC (Ser) resulted in the most embryonic stages with the greatest effect sizes, peaking in E15 (0.44).

In contrast to the liver samples, when we compare eye tissue samples that span from E10 through E18 with strains C57BL/6, C57BL/6J, and C57BL/6N (Supplementary Figures S1G–L), we identify similar numbers of over- and underrepresentation of codons between diseased and healthy genes. We identified 6, 8, and 10 highly overrepresented codons and 9 highly underrepresented codons for the C57BL/6, C57BL/6J, and C57BL/6N healthy eye samples, respectively (7, 7, and 12 DEW codons with RSCU >1.5 and 9, 9, and 10 codons with RSCU <0.5, respectively) (Table 1).

However, between healthy and diseased eye genes, 93% of the synonymous codons for C57BL/6 samples had statistically different RSCU (<0.05), and all the contributing variation was present during E11, E12, and E14 stages (Supplementary Table S3). Effect sizes for C57BL/6 were calculated for all stages, with the majority of the top 10% occurring at E15, showing a large effect for codon GGG (Gly, 9.01) and a very large effect for E18 CCG (Pro, 48.88). In contrast, C57BL/6J and C57BL/6N samples show only 25% of synonymous codon usage, which is significantly different from healthy eye samples.

#### 3.1.2 Fewer codon usage biases are exhibited in diseased and healthy heart genes

Heart samples span stages E9–E18 and belong to strains C57BL/6, C57BL/6J, and CD-1 (Supplementary Figures S1M–R). The healthy and diseased heart-related genes show similarities with liver- and eye-associated genes, but no unique differences were observed within the heart tissue. We identified six highly overrepresented codons and nine highly underrepresented codons for the C57BL/6, C57BL/6J, and CD-1 healthy heart samples (5, 7, and 7 diseased heart codons with RSCU >1.5 and nine codons with RSCU <0.5). C57BL/6 healthy heart samples possess one highly overrepresented codon ACC (Thr) that is not shared with the C57BL/6 diseased heart samples (Table 1). C57BL/6J and CD-1 diseased heart samples are both highly biased toward the codon AGC (Ser), while healthy heart samples are not. All highly underrepresented codons are shared among the healthy and diseased heart samples. In summary, AGC (Ser) was uniquely overrepresented for CD-1 and C57BL/6J diseased heart samples and ACC (Thr) for C57BL/6 healthy heart samples.

We found only a few C57BL/6 (32% with a small effect size for E9, E10, and E12) and C57BL/6J diseased heart samples (36% with a small effect size for some codons in E9 and E13) that were significantly different between healthy and diseased heart RSCU (Supplementary Table S3). The CD-1 heart samples possess codons that are 72% significantly different—most embryonic stages contribute to codon usage variation except for E14 and E17 (small effect size for only the top 10%). Therefore, codon usage biases and codon representation during development vary considerably among the three germ layers and are strain-specific.

### 3.2 Dimension reduction and embryonic clustering interpretations

The RSCU data highlighted the often-significant CUB between healthy and diseased embryonic samples. To further understand these CUB differences, we used the PCA to reduce the dimensionality of our data and identify important codons contributing toward the deviation between healthy and diseased genes. The PCA allows us to visualize how distinct codons are projected (through loading values) onto the sample landscape. With multiple different clustering methods available with different advantages and disadvantages, we were also interested in how clustering methods segregate samples into specific clusters. These analyses aim to determine whether clusters are differentiated by tissue state and whether the codon usage within each cluster is statistically different from one another.

#### 3.2.1 Distinct separation in codon usage and clustering between healthy and diseased liver-associated embryonic genes

**FIGURE 3:**
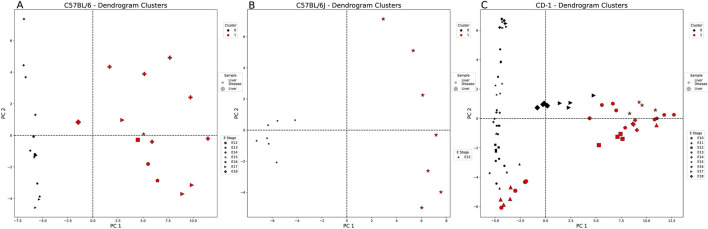
Healthy vs. diseased liver-weighted PCA and clustering example for each embryonic strain. Each figure is labeled with healthy (large markers) and diseased liver samples (small markers). These markers are denoted by the embryonic stage (E) (shape of marker) and the cluster of each sample (cluster 0: black and cluster 1: red). All comparisons are split into two clusters using the agglomerative method. **(A)** C57BL/6 and **(B)** C57BL/6J samples split by sample type (tissue state). **(C)** CD-1 samples are not split by sample type and healthy liver samples from the E17 and E18 groups with the diseased liver samples.

Figure 3 shows liver samples split distinctly by disease association. We observed how codons were projected into the PCA sample space by analyzing the loading values (codon’s contribution to a PC) and individual PC values. From Supplementary Table S4, we identified which codons contribute more significantly to different PCs. In summary, Supplementary Table S5 shows samples with all loading values contributing minimally to PC1. Extreme loading values that fall outside 0.2 and −0.2 will be considered for further discussion. Many codons contributed more heavily to PC2 (≥0.2 or ≤ −0.2; range chosen to highlight the higher-valued codons) for all strains, sharing asparagine (AAT and AAC). Many of the PC2 codons were unique to each strain: C57BL/6—isoleucine (ATC, ATT, and ATT) and phenylalanine (TTC and TTT), C57BL/6J—arginine (CGG and AGA) and proline (CCA and CCG), and CD-1—Gln (CAA and CAG) and Val (GTC and GTT). C57BL/6J and CD-1 share the same heavily contributing codons to PC3 belonging to amino acids—isoleucine (ATA and ATT), phenylalanine (TTT and TTC), and tyrosine (TAT and TAC) (Supplementary Table S5). After identifying numerous codons that contribute to different PCs, we further analyzed them to identify relationships between individual samples and these important codons.

Supplementary Table S6 lists PC values for each sample—a high positive value (>5) for a given PC is interpreted as an important descriptor. Samples with PCs <5 or codons with a loading value between 2 and −2 are not easily interpretable and excluded from further analyses. Interestingly, C57BL/6 PC2 best describes a single diseased E14 liver sample, dominated by A/T-enriched codons when positively correlated (+corr) and T/C-biased codons when negatively correlated (−corr) (Supplementary Table S6). For C57BL/6J samples, PC2 best describes two healthy liver samples with a bias for the C nucleotide, and PC3 best describes one diseased liver sample with a nucleotide preference T>A = C>G. The PCA of the CD-1 samples showed that PC2 best describes eight of the diseased liver samples (all E17 and E18) as A-nucleotide-biased, while PC3 describes six healthy liver samples (half E17 and all E18) as A-nucleotide-biased (+corr) or C-nucleotide-biased (−corr). To analyze these samples more broadly and collectively, we used several clustering techniques.

Four different clustering methods were applied to our PCA dataset with clustering heuristics to help determine method parameters (Supplementary Table S7). If samples are split by tissue state, this reinforces the differences shown in the RSCU comparisons. In this study, we highlight interesting clusters for tissue comparisons.

Liver samples showed the highest similarity metrics when using the agglomerative method. For the C57BL/6 and C57BL/6J samples, all clustering results were split by tissue state (Figures 3A,B). The outbred strain, CD-1, split into two clusters that were mostly defined by tissue state (Figure 3C). The characteristic codons of the mostly diseased liver cluster (black cluster 0—serine and arginine) are not shared with those of the mostly healthy liver cluster (red cluster 1—leucine, threonine, tyrosine, and histidine), and their nucleotide preference is flipped (A = G>C>T vs. T>C>A>G, respectively). All codons were significantly different between the two clusters, except for two arginine codons (AGG and CGC) and a threonine codon (ACG), despite their overlap in sample type (Supplementary Table S8).

#### 3.2.2 Temporal progression in the embryonic stage and tissue state distinction found in C57BL/6N samples

C57BL/6, C57BL/6J, and C57BL/6N strains spanning E10–E18 demonstrated that eye samples split by tissue state with some overlap. Unlike liver tissue, in the eye, embryonic stage progression is less obvious. Supplementary Table S5 shows C57BL/6J codons contributing heavily to PC1 [cysteine (TGT and TGC), histidine (CAT and CAC), and phenylalanine (TTT and TTC]—mainly diseased E14 and E15 eye samples {bias toward C (+corr) and T nucleotides (−corr)}], unlike C57BL/6 and C57BL/6N samples (Supplementary Table S4). The majority of C57BL/6 E15 and E18 diseased eye samples (+corr: C nucleotide, −corr: C/G) and C57BL/6J healthy and diseased eye E17 samples (+corr: A, −corr: G) share glycine (GGA and GGC) for PC2. Additionally, C57BL/6 [primarily E16 samples (+corr: T/C, − corr: G) and C57BL/6J (mostly diseased eye samples from E17 (+corr: C, −corr: G)] share the serine codon TCT and uniquely possess TCG and TCC for PC3, respectively (Supplementary Table S6). Across all weighted samples, liver samples tend to use codons with A/T nucleotides (+corr) and T/C nucleotides (−corr); in contrast, the eye samples tend to use codons with C (+corr) and G (−corr).

**FIGURE 4:**
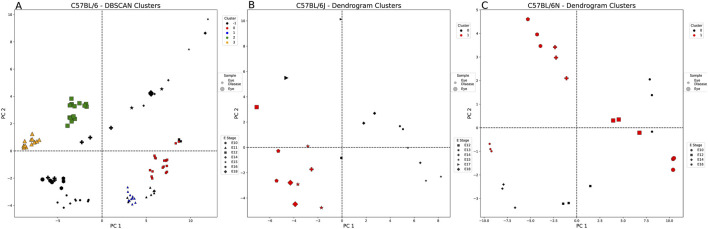
Healthy vs. diseased eye-weighted PCA and clustering example for each embryonic strain. Each figure is labeled with healthy (large markers) and diseased eye samples (small markers). These markers are also denoted by the embryonic stage (E) (shape of marker) and the cluster of each sample. **(A)** DBSCAN split the C57BL/6 samples (E10–12, E14–16, and E18) by sample type (tissue state), excluding three of the healthy eye samples from E12 (black: outliers, red: cluster 0, blue: cluster 1, green: cluster 2, and orange: cluster 3). **(B)** The dendrogram split the C57BL/6J samples (E12–15 and E17–18) by sample type, except for one healthy eye sample from E17 (black: cluster 0 and red: cluster 1). **(C)** The dendrogram split the C57BL/6N samples (E10, E12, E14, and E16) by sample type, except for the diseased eye samples from E16 (black: cluster 0 and red: cluster 1).

Supplementary Table S7 provides a summary of the clustering evaluation metrics for each of the eye-weighted strains. For C57BL/6 (Figure 4A), DBSCAN clustering assigned four clusters, which were split by the tissue state and embryonic stage: 1) healthy E11, 2) healthy E12, 3) diseased E11, and 4) diseased E12 eye samples. These clusters highlight the potential importance of CUB in the E11 and E12 stages of embryonic eye development. Cluster 3 (orange—healthy eye E11) is slightly more similar in codon usage to cluster 1 (blue—diseased eye E11) than to cluster 2 (greenhealthy eye E12). Figure 4 presents a different clustering method listed for panel A compared to panels B and C. We chose to highlight only the clustering methods with the highest clustering metrics (see *Methods*). Figure 4B shows that the C57BL/6J dendrogram split samples into two clusters—one healthy E17 eye sample clustered with the diseased eye samples, although many codons were significantly different (Supplementary Table S8). The diseased eye cluster (black) was AA enriched for isoleucine and asparagine, and the healthy eye cluster (red) was enriched for arginine and aspartic acid, although both were similarly biased toward A and against T nucleotides. C57BL/6N samples, as shown in Figure 4C, split into two mixed clusters: 1) cluster 0 (black)—all diseased eye samples except E16 and 2) cluster 1 (red)—all healthy eye and E16 diseased eye samples. Cluster 0 characteristic codons are AA enriched for arginine and are biased toward C>T = G = A nucleotides (Supplementary Table S4). Cluster 1 is not biased for any AAs and has a nucleotide bias T>C>A>G, suggesting that, unlike diseased eye genes, healthy embryonic eye development exhibits less codon usage preferences.

#### 3.2.3 Large codon usage variance found in healthy and diseased heart genes

**FIGURE 5:**
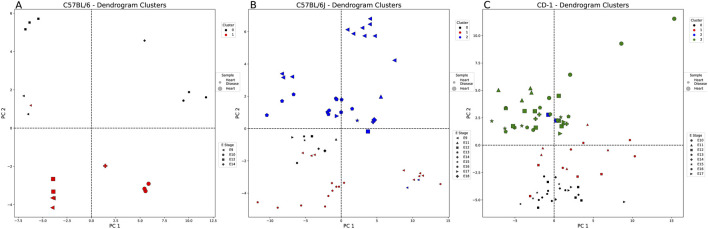
Healthy vs. diseased heart-weighted PCA and clustering example for each embryonic strain. Each figure is labeled with healthy (large markers) and diseased heart samples (small markers). These markers are also denoted by the embryonic stage (E) (shape of marker) and the cluster of each sample (cluster 0: black, cluster 1: red, cluster 2: blue, and cluster 3: green). All comparisons were split into clusters using the agglomerative method. **(A)** C57BL/6 samples (E9–10, E12, and E14) split by sample type (tissue state), except for one of the E9 diseased heart samples. **(B)** C57BL/6J samples (E9 and E11–18) split by sample type, except for two E9 diseased heart samples. **(C)** CD-1 samples (E10-17) are split by sample type into four clusters.

This comparison includes strains C57BL/6, C57BL/6J, and CD-1 spanning E9 through E18. Figure 5 shows that C57BL/6J and CD-1 diseased heart samples lie in the positive PC2 quadrants and C57BL/6 in the negative PC2 quadrants. None of the strains show any distinct embryonic stage patterning. Supplementary Table S5 shows that CD-1 samples possess several codons contributing heavily to PC1, predominantly from aspartic acid and glutamic acid. There is no overlap in the strains’ AAs for PC2 or PC3. CD-1 heart-weighted samples show codons related to arginine and leucine for PC4.

In addition, E9–E12 seem to be critical periods of heart development that can be separated through PCA clustering across strains. CD-1 samples were split across four PCs with varying nucleotide preferences dependent on codon correlation. CD-1 samples show several codons contributing more heavily to PC1 (≥0.2 or ≤ −0.2), predominantly from aspartic acid (GAT and GAC) and glutamic acid (GAA and GAG). Supplementary Table S4 shows that C57BL/6 diseased E12 heart samples best describe PC2 with a codon preference toward C (+corr) and T nucleotides (−corr) (also observed in C57BL/6J healthy E9 heart samples). However, this similarity in nucleotide preference does not directly translate into the same preferred codons. Supplementary Table S6 shows that there is very little overlap in the strains’ amino acids for PC2. No amino acids are shared between the C57BL/6J (arginine—AGG and CGA; leucine—TTA and CTC) and CD-1 (serine—TCC and TCT; threonine—ACC and ACT; valine—GTG and GTT) samples for PC3. CD-1 has no other strain comparison for PC4 but shows many heavily contributing codons, predominantly from arginine (AGG and CGA), leucine (CTG and CTC), and serine (TCC and AGC). Combining all strains, tissue states, and embryonic stages shows that the heart-weighted samples are slightly more similar to the eye-weighted samples than the liver-weighted samples in that they more often possess codons with C nucleotides when positively correlated. In contrast to both the liver- and eye-weighted samples, the heart-weighted samples tend to be C-dominated codons when negatively correlated.

#### 3.2.4 Greater codon usage separation between healthy and diseased heart genes in C57BL/6 samples

The agglomerative clustering method grouped the C57BL/6 samples clearly by disease association (Figure 5A). The diseased heart cluster’s (black cluster 0) characteristic codons are not overly influenced by any AAs (C>A>T>G). In contrast, the mostly healthy heart-weighted genes (red cluster 1) are heavily influenced by serine, threonine, and arginine AAs and biased toward C>G>T>A. For CD-1 in Figure 5C, when comparing the diseased heart clusters [cluster 1 (red) and cluster 3 (green)], all codons are significantly different, except for those primarily belonging to glycine, leucine, and arginine (Supplementary Table S8). If we take a closer look at the characteristic codons of the diseased heart samples, cluster 3 (green) is AA-enriched for valine, leucine, alanine, phenylalanine, asparagine, and tyrosine [compared to cluster 1 (red)] and biased for C>T>G>A [cluster 1 (red): G>C>A>T].

## 4 Discussion

In this study, we investigated CUB dynamics based on comparisons of liver-, heart-, and eye gene-weighted RSCU for four murine strains across many embryonic stages (Fumagalli et al., 2024) [Mouse Embryo CoCoPUTs (https://dnahive.fda.gov/hivecuts/mouse_embryo/)]. Our work uncovered many significant differences in codon usage across tissue-specific weighed samples, which can be separated based on disease association, unique strains, and embryonic stages (Supplementary Table S2). Among these comparisons, we observed the following: 1) CUB patterns vary among individual tissue groups, most notably with the eye having the greatest RSCU variation and the liver having the least, and 2) disease-associated genes show distinct CUB relative to healthy genes in embryonic development.

We demonstrated that these tissue developmental differences in RSCU persist across all tissue types with slight differences in over- and underrepresented codons and variation/identity of CUBs. These differences in codons encoding AAs can have a significant impact on gene expression patterns, affecting developmental processes. For example, arginine, one of many codons we identified to have significant CUB, is critical for embryonic survivability (Wu et al., 2013). AGA (Arg), along with glycine, is the first rate-limiting step in creatine synthesis, which influences the embryonic development of neurological and skeletal muscle. Single-gene disorders are also linked to the use of CGN codons over AGG or AGA (Rossi et al., 2022; Schulze et al., 2020). Interestingly, CGT (Arg) usage is highly variable among human tissues like skin, muscle, and kidney, with a large overrepresentation in muscle disease-causing genes (Rossi et al., 2022). Conversely, neurodegeneration and cancer are linked to an overrepresentation of AGG (Arg) and an underrepresentation of CGT (Arg) (Khandia et al., 2023).

In this study, we found that CGC was preferred in all healthy and diseased eye and healthy heart CD-1 samples, but CGG usage was preferred in the CD-1 diseased heart samples. Liver-weighted strains, C57BL/6, C57BL/6J, and CD-1, showed variation in preferred codons—AGG, AGA, and AGG, respectively. In comparison, variation was only found between tissue states for the least-used codon (CGA in healthy samples and CGT in diseased samples). The eye-weighted samples show no variation within or between sample types for the most preferred codon (GCG). However, there is some variation within and between sample types found for the least-preferred codon (C57BL/6 and C57BL/6J healthy and diseased eye: CGT; C57BL/6N healthy eye: CGA; C57BL/6N diseased eye: CGC). The heart-weighted samples revealed variation in arginine usage both within and between tissue states for the most preferred codon (C57BL/6 and C57BL/6J healthy and diseased heart: AGA; CD-1 healthy heart: CGC; CD-1 diseased heart: CGG), but no variation was observed in the least-preferred codon (CGT for all strains and tissue states). These observations suggest that different germ layers may require certain codon usage biases, such as for the critical AA arginine, to support the expression of genes important for healthy development.

Furthermore, important observations were the differences in distinct codon signatures across embryonic mouse strains, potentially demonstrating changes in their overall differentiation patterns and highlighting the selectivity required for choosing strains for testing preclinical therapeutics (Chebib et al., 2021; Keane et al., 2011). The average CUB of the C57BL/6 samples deviated from that of other strains when weighted by healthy liver genes—underrepresenting: CTT (Leu), TCA (Ser), and GTT (Val). C57BL/6J diseased liver samples uniquely underrepresent GCT (Arg). We also found a divergence in the C57BL/6N strain when weighted with diseased eye genes—overrepresented: CTC (Leu), TTC (Phe), and CCC (Pro) and underrepresented: GTT (Val). Interestingly, in contrast to liver and eye tissues, the heart showed no differences in over- and underrepresented codons across strains when weighted by both healthy and diseased-associated genes. As different tissues each have their unique gene expression patterns, it is possible that different germ layers may have different temporal embryonic gene expression needs and may be represented by differences in CUB. Although organ tissues may develop similarly phenotypically across different murine strains, organogenesis may differ, with changes based on the strain and environment. The strain response to injury, drugs, and disease processes may vary and influence murine models of congenital diseases compared with humans (Sellers, 2017).

When we look at human genomic RSCU, codons CTG (Leu), GCA (Ala), and GTG (Val) are overrepresented and TCG (Ser), GCG (Ala), CGT (Arg), CTA (Leu), CCG (Pro), and ACG (Thr) are underrepresented (Supplementary Table S9) (Kames et al., 2020). Interestingly, compared to our mouse embryo data, we found that GCA (Ala) is neither overrepresented in any of our strains nor in tissues, and CGT (Arg) is only underrepresented in strain C57BL/6J for the liver. Humanized mouse models are one approach attempting to tackle codon usage differences across strains and tissues. For example, modifying three specific murine codons in the amyloid precursor protein (APP) gene reconstructs the condition necessary for the development of Alzheimer’s disease (Reaume et al., 1996). To study sickle β -thalassaemia in mice, knockout of the adult β -globins failed to create what had been observed in human patients; however, the replacement of the mouse β *globin* genes with human codon bias improved postnatal survival (Huo et al., 2009). We note, however, that the relationship between CUB and gene expression is rather complex. Codon usage is known to affect exonic transcription factor binding and transcription efficiency, mRNA splicing, biogenesis and stability, the efficiency/stringency of mRNA decoding, and finally protein biogenesis and folding (Komar, 2016; Wu and Bazzini, 2023; Bailey et al., 2021; Liu et al., 2021; Moss et al., 2024). Future studies analyzing gene expression data and CUB relationships will provide insight into the exact underlying mechanism and potential differences in individual germ layer development across various strains.

To reduce codon complexity, we used PCA to compile the variance of 59 codons and then project the codons (features) into our sample space. We identified potential critical embryonic periods that may be important temporal points in development, requiring certain codon usage preferences. Among many other specific periods discovered, we identified E15 as a potentially critical period for liver-associated genes as disease genes separated most clearly from healthy genes. Elevated expression of the *Lxts1* gene has been shown during E15, and mutation can lead to hepatocellular carcinoma (Mu et al., 2020). Further analysis is required to determine whether specific timed sequences of gene expression, which are essential for determining cell fate, are present at the codon level during embryogenesis. These temporal relationships likely require a deeper analysis of individual high- or low-expressing genes.

Important AAs per strain were defined as having a PCA codon loading value of ≥0.2 or ≤ −0.2 and possessing at least two synonymous codons that met this criterion. Under these assumptions, a strain weighted by a specific set of genes tends to show uniquely critical AAs (Supplementary Tables S2, S4, S6, S8). Of these AAs and across all gene-weighted comparisons, C57BL/6 and C57BL/6J samples were more often associated with nonpolar AAs, C57BL/6N samples with polar AAs, and CD-1 samples with equally nonpolar and negatively charged AAs. Many of the comparisons revealed quite a bit of difference in PC heterogeneity—making it difficult to relate specific codons to whole tissue state groups. Nonetheless, PCA analysis was able to reveal unique codon preferences for individual strains and highlighted the codon usage differences between diseased and healthy tissues.

To further capture better and more insightful patterns in CUB based on changes to embryonic stages, strains, and tissue states, we used a set of clustering methods, evaluation metrics, and the known biology of the samples to focus further analyses. This revealed that clustering methods resulting in the highest evaluation metrics often produced clusters with the most significantly different codons. There were a few examples of clusters splitting strictly by tissue state. C57BL/6 healthy and diseased liver clusters resulted in 95% of their CUB being significantly different and both preferred nucleotide C. C57BL/6J healthy and diseased E15 liver clusters showed that 80% of their codon usage was significantly different, with their nucleotide preferences completely flipped from one another. In another case, CD-1 healthy and diseased heart clusters were split into four, and diseased heart clusters were independently shown to be closer in CUB to the healthy heart clusters than to each other. The results of these clustering analyses highlight the need for further investigation into other sources of variation (i.e., GC content and codon pair bias) that might contribute to these clusters.

Thus, in this study, we demonstrated that CUB patterns differ across embryonic development between strains, tissue types, and tissue states. We highlighted critical embryonic stages that showed significant deviations in CUB preferences within individual tissues. Further pairing of temporal gene expression data with this codon usage analysis may help elucidate the biological relationships mediated by gene expression that are important for embryogenesis. These data are also of interest for comparing tissue-specific CUB between embryonic and adult tissues and describing how CUB changes during embryogenesis impact genes that influence liver cancer or liver fibrosis later in life. Future studies evaluating different mouse strains, especially developmental studies spanning multiple embryonic stages or strain targeting for the pre-clinical testing of therapeutics (e.g., mRNA-based), should be aware of the impact of these CUB differences. These findings are critical for understanding the relationship between codons and embryonic development stages and provide the necessary biological context for future studies evaluating disease gene expression relationships across development.

## Data Availability

Publicly available datasets were analyzed in this study. These data can be found at: https://dnahive.fda.gov/hivecuts/mouse_embryo/.
