# Supplementary figures and images for: Uncovering codon usage patterns during murine embryogenesis and tissue-specific developmental diseases
Source: Front Genet. 2025 May 26;16:1554773. doi: 10.3389/fgene.2025.1554773 (PMC12146342; doi:10.3389/fgene.2025.1554773)

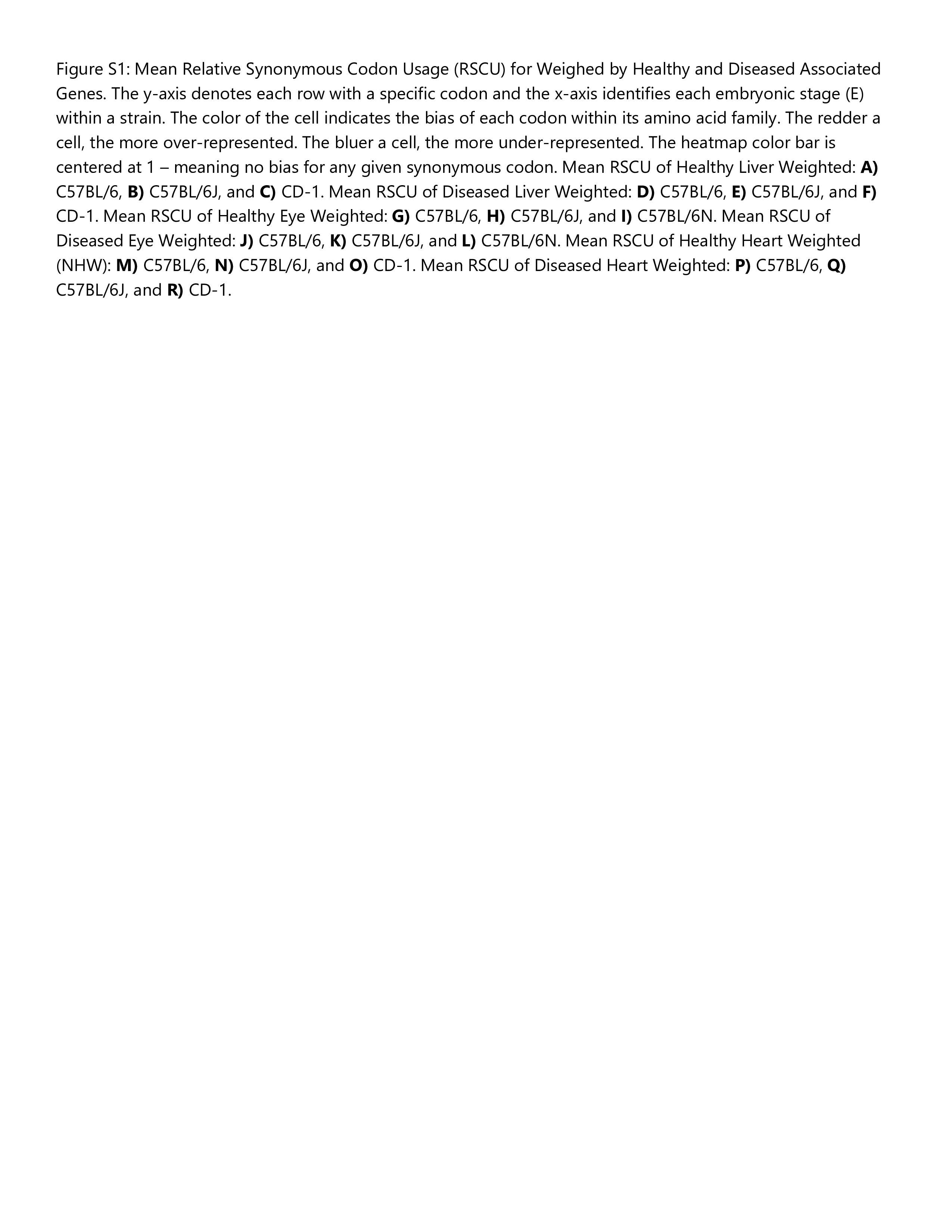

Supplement: Supplementary file 4 [file DataSheet1.zip › 884dfc3f-7724-4d56-af26-1ee5fa5e382d-0.jpg]

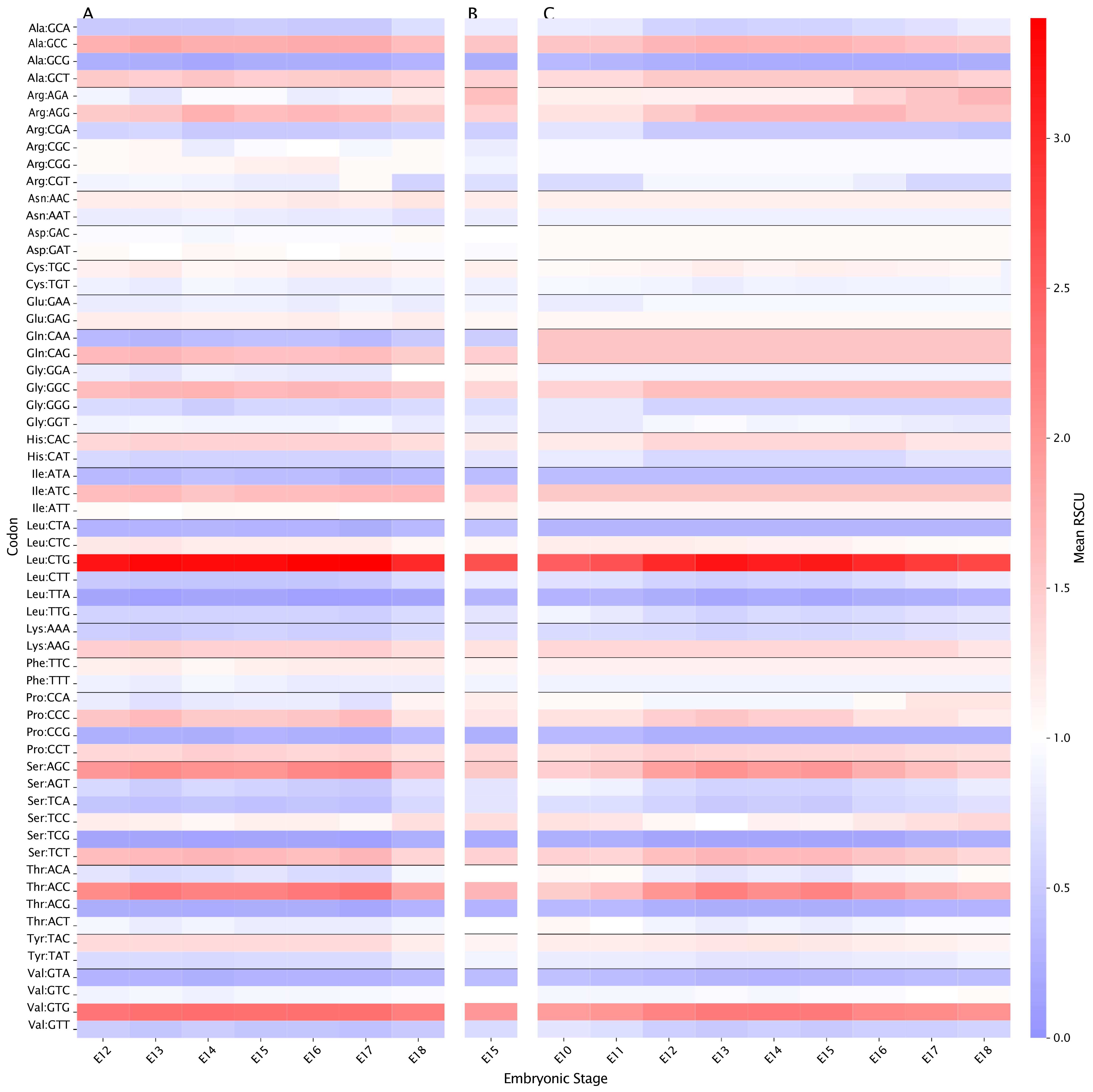

Supplement: Supplementary file 4 [file DataSheet1.zip › 884dfc3f-7724-4d56-af26-1ee5fa5e382d-1.jpg]

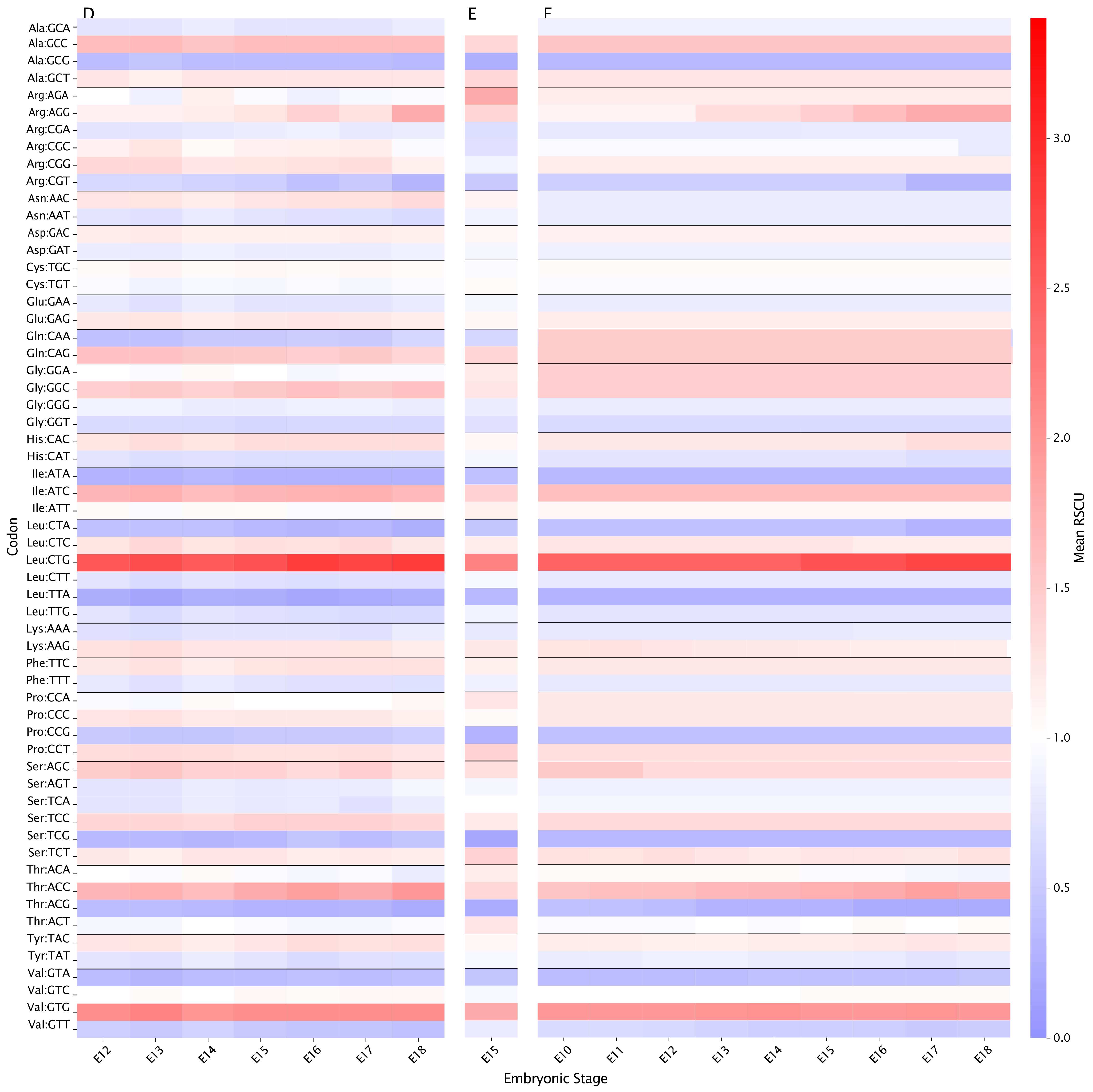

Supplement: Supplementary file 4 [file DataSheet1.zip › 884dfc3f-7724-4d56-af26-1ee5fa5e382d-2.jpg]

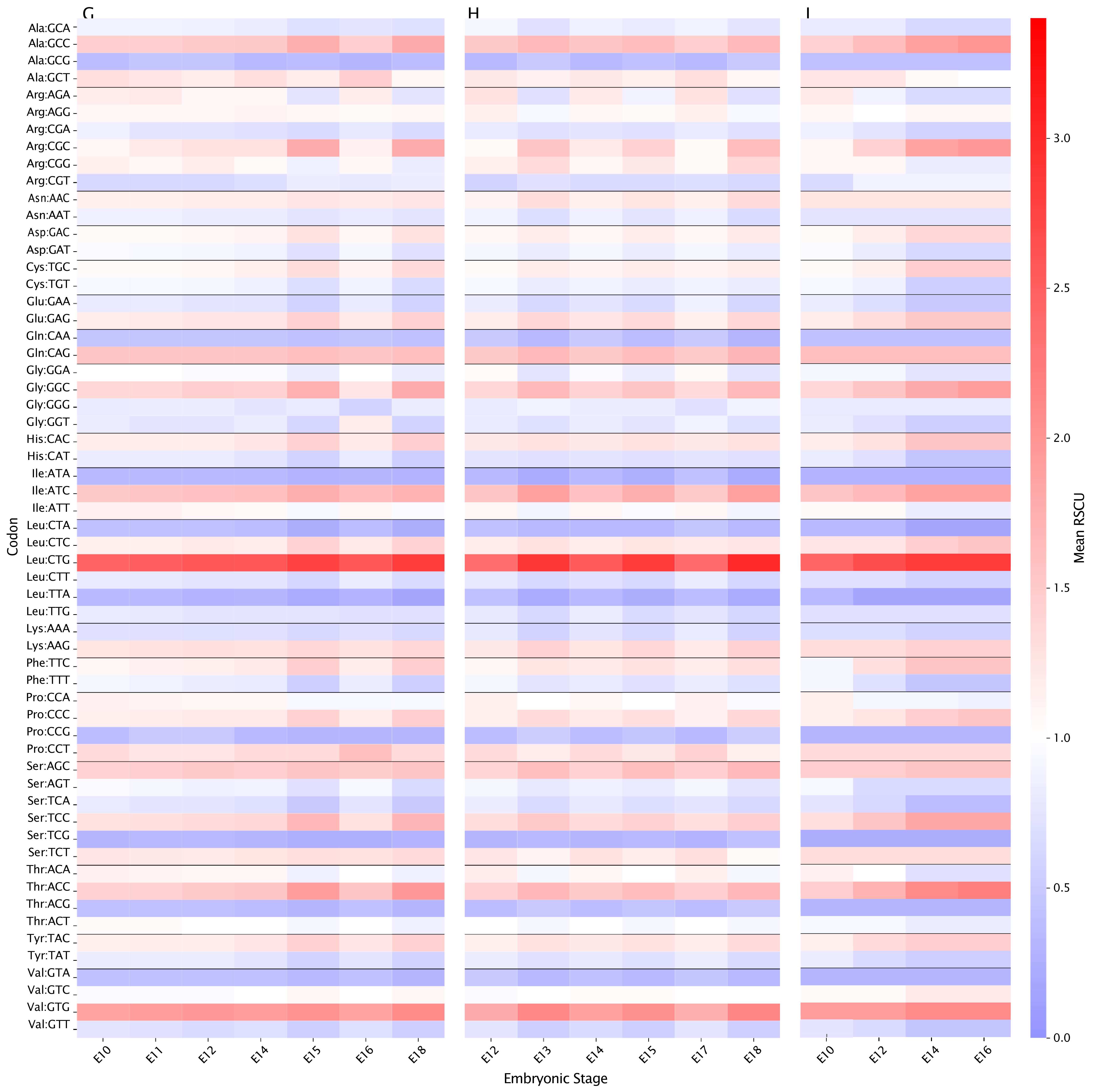

Supplement: Supplementary file 4 [file DataSheet1.zip › 884dfc3f-7724-4d56-af26-1ee5fa5e382d-3.jpg]

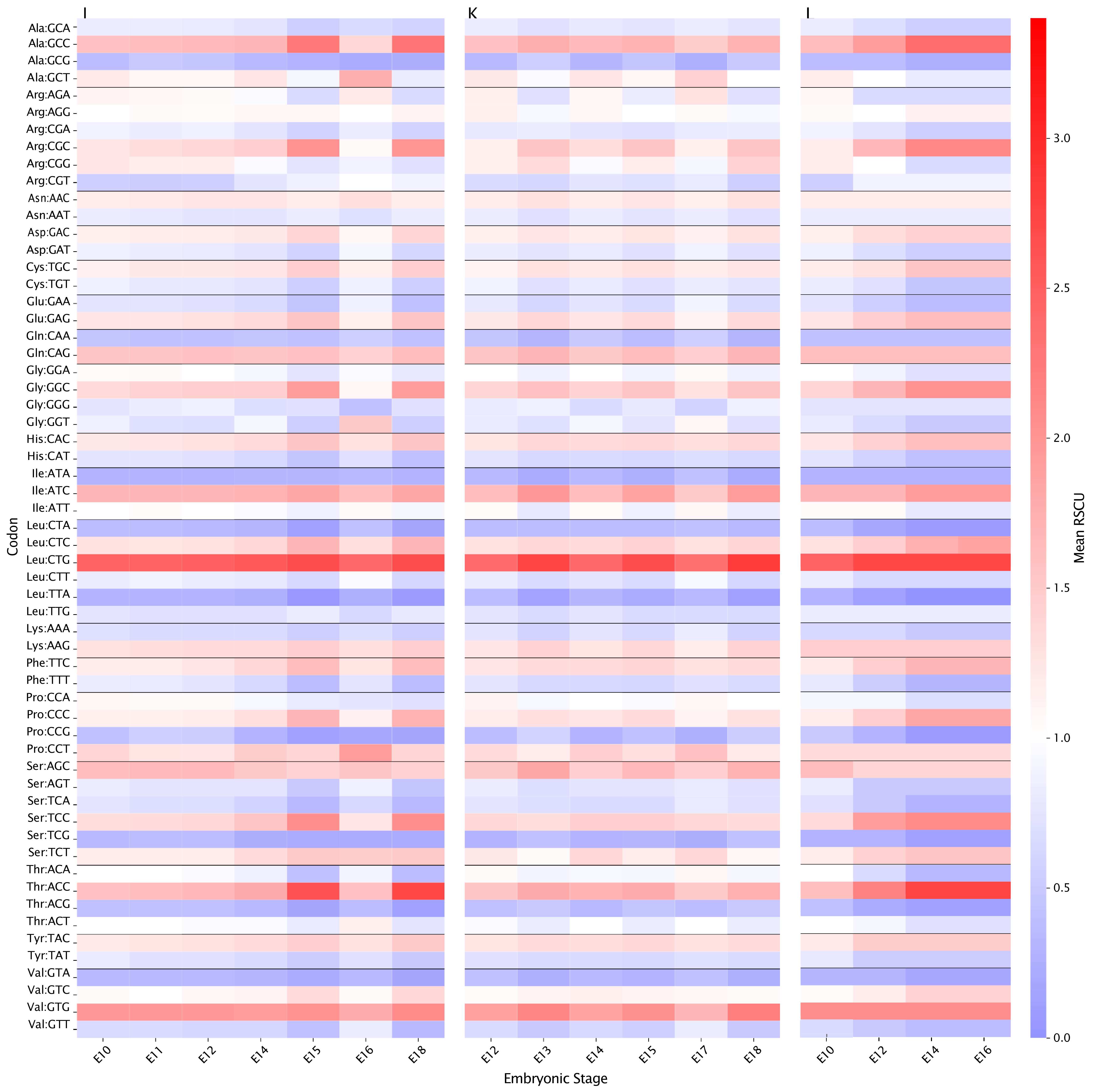

Supplement: Supplementary file 4 [file DataSheet1.zip › 884dfc3f-7724-4d56-af26-1ee5fa5e382d-4.jpg]

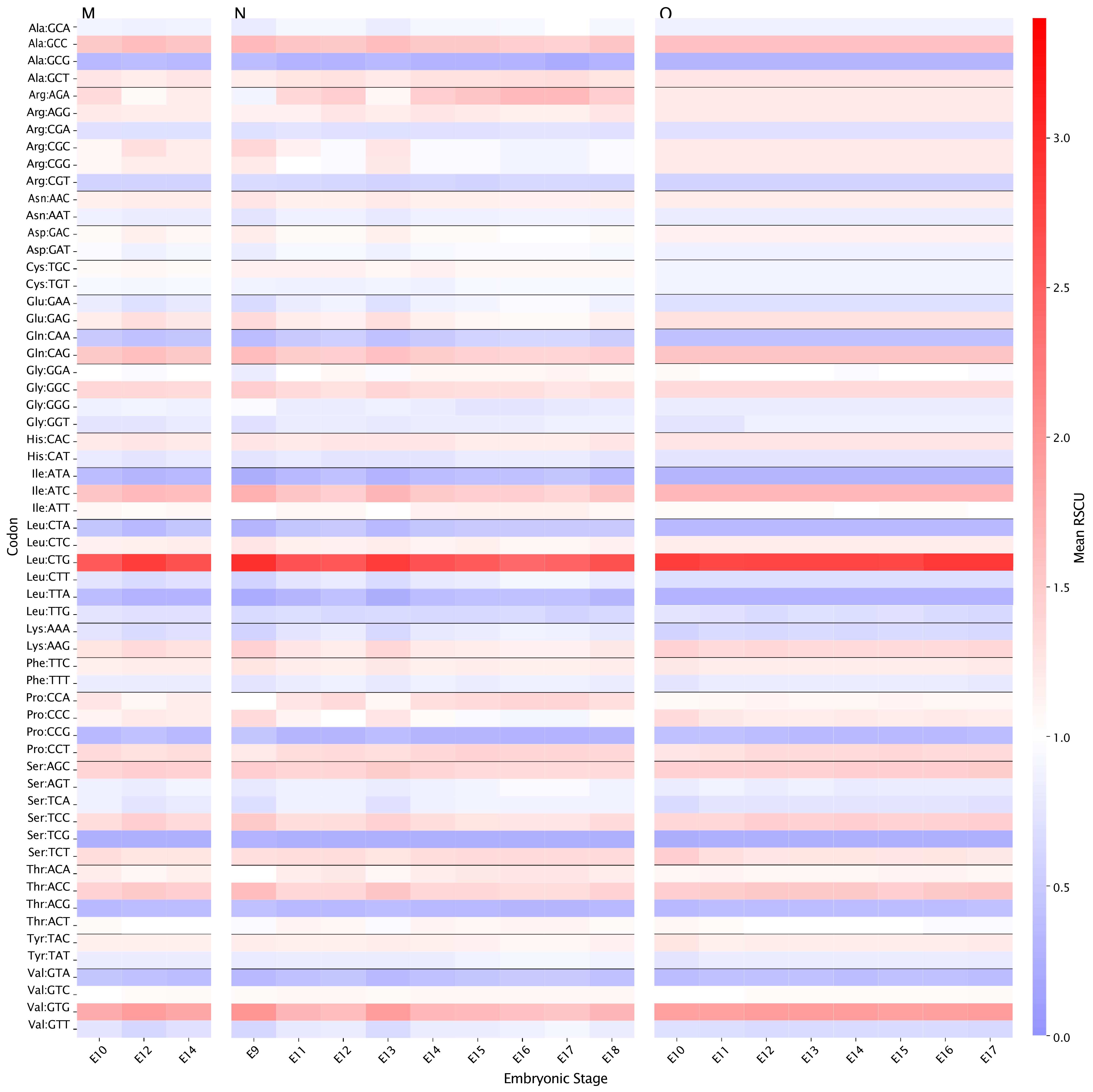

Supplement: Supplementary file 4 [file DataSheet1.zip › 884dfc3f-7724-4d56-af26-1ee5fa5e382d-5.jpg]

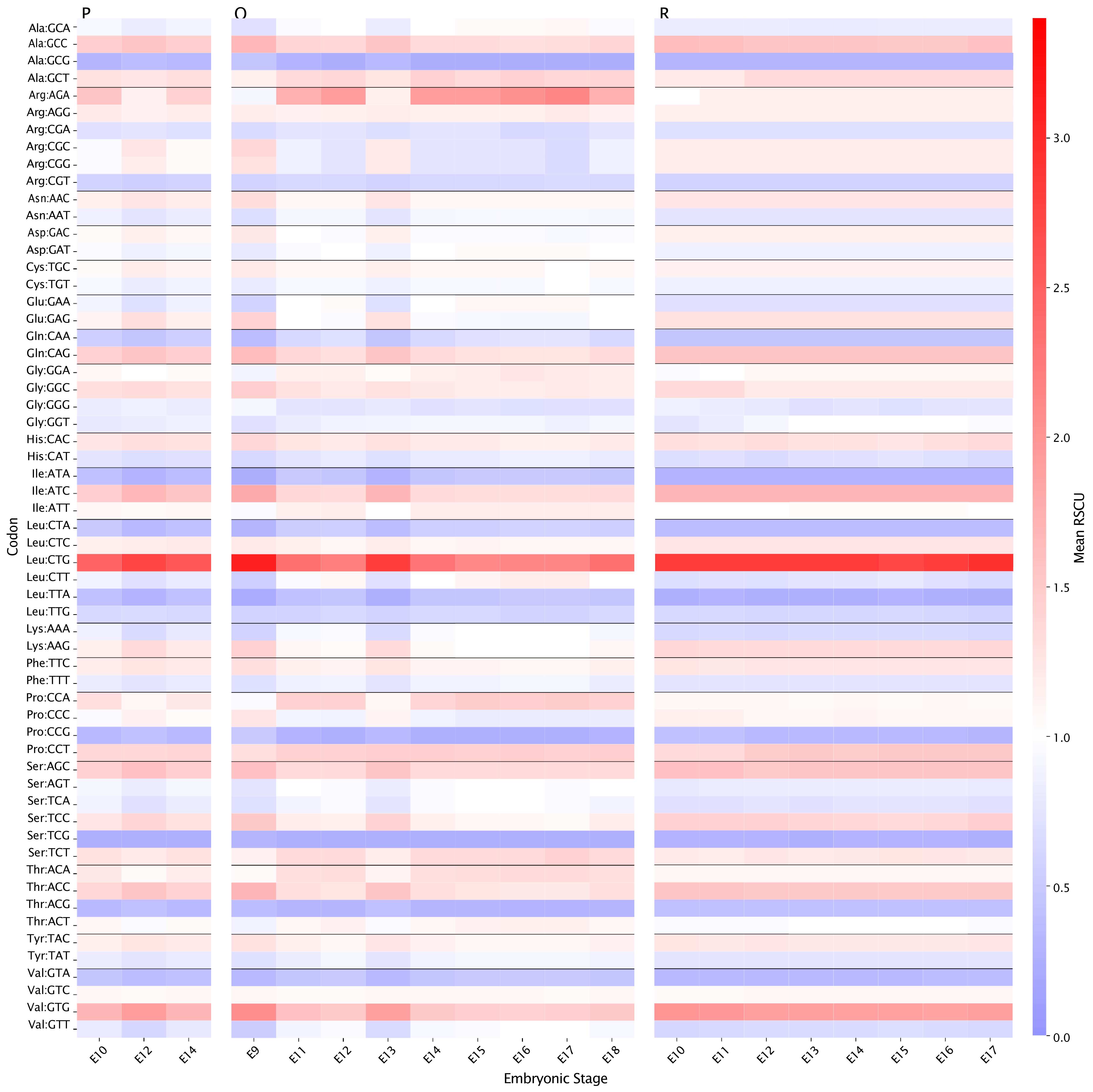

Supplement: Supplementary file 4 [file DataSheet1.zip › 884dfc3f-7724-4d56-af26-1ee5fa5e382d-6.jpg]
